# A qualitative exploration of staff satisfaction in innovative Australian aged care

**DOI:** 10.1111/ajag.70090

**Published:** 2025-09-08

**Authors:** Suzelie G. Connelly, Prudence Millear, Kirsten Tulloch

**Affiliations:** ^1^ School of Health University of the Sunshine Coast Sippy Downs Queensland Australia

**Keywords:** job satisfaction, organisational innovation, qualitative research, residential aged care facility

## Abstract

**Objectives:**

Long‐term worker shortages in Australian residential aged care are well‐documented. These shortages adversely impact residents' well‐being and the morale of staff caring for them. This study aimed to explore staff and management experiences through workplace theories related to worker satisfaction: job demands‐resources theory, self‐determination theory, moral disengagement and work as calling theory, at NewDirection Care, which provides innovative aged care in Queensland. A further aim was to identify the impact of the innovations on staff satisfaction.

**Methods:**

Semi‐structured staff and management interviews (*n* = 18, 13 females, 5 males) were held on‐site. An appreciative inquiry approach identified the under‐researched linkages between staff satisfaction in innovative aged care and workplace theories. The NVivo 12.0 qualitative analysis software was used for reflexive thematic analysis of interviews.

**Results:**

Three themes emerged from the interviews: (1) the supportive management culture, (2) the innovative conditions and (3) finding meaningfulness at work. These themes demonstrated how staff attitudes linked to the chosen workplace theories and identified how specific innovations, combined in both the physical environment and models of care, were associated with improved staff satisfaction.

**Conclusions:**

The current study indicates that workplace initiatives may increase staff satisfaction, potentially ameliorating shortages by attracting and retaining staff. Semi‐structured interviews identified that specific innovations, combined in both the physical environment and models of care, are associated with improved staff satisfaction. Reflexive thematic analysis of interviews contributed to the literature and suggested future research opportunities, such as examining differences in aged care worker satisfaction between traditional and innovative residences.

**Practice impact**

To improve retention of staff through increased staff satisfaction, there are opportunities for traditional aged care providers to innovate in line with workplace theories. Innovations could focus on the development of a moral leadership ethos, with an emphasis on the individual dignity of residents and employee autonomy.

**Policy impact**

The study found that this combination of specific residential aged care innovations was associated with increased staff satisfaction. Further research into innovations and how they may be combined to augment staff satisfaction may result in greater retention of staff in the aged care sector.

## 1 INTRODUCTION

Staffing levels in residential aged care homes remain an ongoing concern in Australia. Although there are currently 360,000 aged care staff, current staffing shortfalls (35,000) and predicted shortfalls (110,000 by 2030 and 400,000 by 2050) impact the provision of minimum standards of care.^1^ The percentage of Australians aged over 65 years is predicted to rise from 16% currently to 23% by 2066, increasing the numbers of older Australians likely to need care in the future.^2^ In this context, finding and retaining suitable staff is crucial for the aged care system, whilst also taking pressure off hospitals as alternative places of care.^1^ The recent Royal Commission into Aged Care Quality and Safety (the Royal Commission)^3^ found that high attrition of personal care workers had deleterious flow‐on effects, including lowered standards of care and increased mortality for older adults in residential aged care homes.^3^

Providing person‐centred care (PCC) is the foundation of quality aged care. Higher levels of PCC have demonstrated benefits for residents and are more likely where staffing levels are sufficient, staff have more time for residents, and the aged care environment is more supportive of residents.^4^ Barriers to care for staff in traditional residential aged care homes include dissatisfaction with heavy workloads, undervalued contributions, under‐resourcing, wages disparity, and in particular the reduced time to provide holistic, meaningful care.^5^ A lack of understanding of PCC, insufficient time for care and changed behaviours of residents can also limit the implementation of PCC.^6^ The focus of the current study is to understand how innovation in models of aged care may increase retention of staff, increase staff job satisfaction and create the organisational structures that provide staff with resources to deliver more PCC.

Innovative aged care approaches to enhance PCC are being implemented in Australia and internationally. For instance, in the Netherlands, over 1000 green‐care farms are transforming aged care.^7^ These innovations have been studied extensively. Green‐care farms combine PCC in non‐institutional aged care homes with agricultural pastimes.^7^ Quantitative cross‐sectional studies of people living with dementia across different types of aged care (*n* = 115) identified the advantages for residents in innovative, compared with traditional, aged care and found that green‐care farms provided tailored care that resulted in higher scores on quality‐of‐life indices.^7^

A recent scoping review^8^ noted the challenges of working in residential aged care homes were increased by a compliance culture. Meeting complex regulatory requirements exacerbated the challenges of physically and psychologically demanding work, and were compounded by adequately meeting the needs of ethnically and linguistically diverse staff and residents.^8^ Innovations in delivery of aged care may incentivise staff to remain in the sector, addressing the current staff shortfalls. Understanding organisational structures that support innovation and high levels of PCC^6^ and staff experiences^4^ in these workplaces are likely to provide the basis for strategies to reduce turnover, offset the burden of government regulation, overcome recruitment difficulties, and increase job satisfaction and engagement.

Several theoretical frameworks can explore how innovative models of care may be used to implement high levels of PCC in residential aged care homes. Firstly, the Job Demands‐Resources theory (JD‐R)^9^, ^10^ can explain the structure of work (at all levels of an organisation). All jobs, including those of employees in caring roles (such as nurses), have demands from what is to be done (e.g. emotional demands, workload and time pressure) and resources to manage those demands (including job autonomy, social support and leadership). Where demands exceed available resources, burnout and turnover tend to occur, whereas more resources to manage demands increase motivation and work engagement.^9^, ^10^ Staffing issues were identified by a scoping review,^8^ that compared low versus high PCC.^4^, ^6^ Low PCC may be understood through the presence of greater job demands for staff, which reduce the quality of care. In contrast, higher levels of PCC may reflect greater resource availability to staff, which is shown by a positive workplace culture and leadership ethos, sufficient staff, organisational pride and collaborative teamwork.^4^, ^8^

Second, the structures of intrinsic motivation and autonomous extrinsic motivation are described by self‐determination theory (SDT), which explains how self‐determination satisfies human psychological needs of autonomy, competence and relatedness.^11^, ^12^ Both JD‐R and SDT propose that job resources intrinsically motivate staff through fulfilling psychological needs, such as job autonomy, social supports and positive feedback.

Finally, moral disengagement (MD)^13^, ^14^ and work as calling theory (WCT)^15^, ^16^ focus on work as morally good or as a vocation. Applied to aged care, high‐quality standards of care are driven by personal values and beliefs, protecting against cynicism and diffusion of responsibility. As such, motivation at work is also influenced by vocational striving and by moral values, which maintain moral engagement for staff.^13^, ^14^, ^15^, ^16^

Provision of high PCC levels and retention of suitably motivated and committed staff in residential aged care homes is an ongoing concern for governments, providers and users of aged care.^1^, ^2^, ^3^ The current study was conducted in an innovative aged care environment in South‐East Queensland, NewDirection Care, which has incorporated organisational changes, purpose‐built features, staff role changes and targeted recruitment of staff in an innovative model of care. The current study explored, firstly, staff experiences of the workplace through the proposed theoretical frameworks to understand whether these factors (e.g. more job resources, increased intrinsic motivation, support for meaningful and valuable work) increased job satisfaction. Secondly, the study explored how working conditions in residential aged care homes may be improved by the built environment and models of care. Such innovations may potentially be useful to attract and retain a skilled and dedicated workforce. We explored the fit between theory and the lived experience of staff to investigate the research question, *how are the innovations at NewDirection Care associated with staff satisfaction, and by what theoretical pathways?*

## 2 METHODS

### 2.1 Participants

This study was approved by the Human Research Ethics Committee, University of the Sunshine Coast (Ref: S241972). Volunteers (*n* = 18, 13 females, 5 males) were recruited from current members of general staff (*n* = 6), personal care workers (*n* = 6) and those in management roles (*n* = 6). Theoretical sampling was undertaken to ensure participants were representative of age, gender, experience and role types amongst the overall staff (*n* = 140). Ages ranged from young adults to mature‐aged persons. Inclusion criteria were English comprehension and current employment at NewDirection Care for at least one shift a week (general roles, personal care workers) or for 2 days a week (management roles).

### 2.2 Setting for research

At NewDirection Care, the work environment is situated within a *specific organisational context* (as policies and procedures, and the shared ethos of all staff in the organisation). The work is enacted in a *unique built environment*, in 17 seven‐person homes that are not segregated by diagnosis or physical condition. Extensive facilities as a MicroTown™ (trademark of the organisation) mimic everyday community life, with gardens, walkways and shopping areas, which include a corner store, café, hairdresser and medical facilities (a wellness centre and rooms for a dentist and allied health professionals). Finally, the care of residents is undertaken as *redesigned and bespoke work roles*, as descriptions and expectations for roles. Examples are the newly designated role of House Companion™ (trademark of the organisation), and greater expectations of interactions with residents, regardless of work role, which link together to deliver more person‐centred care for residents.

### 2.3 Research design

This was a qualitative, cross‐sectional design, which used appreciative inquiry with a constructivist approach.^17^ Research design was based on the adopted theories outlined above, as being likely to support explanations for staff satisfaction.

### 2.4 Measures and interview questions

Interview questions explored working conditions, previous aged care experiences and motivations for current work roles, to understand how innovative models of care affected employee commitment, satisfaction and likely retention. Questions were similar between management and general staff roles. Sample questions included, ‘Is there something in particular that you think makes residents and staff happy?’, and ‘What do you tell other people when they ask about your experiences working here?’

### 2.5 Procedure

Convenience sampling was undertaken, with information about participation provided to all NewDirection Care staff through fliers displayed in staff areas, with a QR code used to register interest. All participants provided informed consent prior to inclusion in the study. The organisation allowed staff to complete interviews during worktime and emphasised voluntary involvement to increase the reliability and authenticity of respondents' comments.^18^ Data were collected from volunteers in semi‐structured, individual, in‐depth interviews to examine staff and management experiences, continuing until data saturation occurred (when no additional insights were provided).^19^ Interviews (*n* = 18) were conducted in private spaces, either in person (*n* = 13) using the first author's personal motorhome parked adjacent to NewDirection Care, or by Zoom (*n* = 5), lasting on average 23 min. Participants were offered review of transcripts, although no one accepted.^20^ Interviews were digitally recorded, transcribed verbatim (using Otter.ai), manually checked and then deidentified of personally identifying information.

### 2.6 Data analysis

Transcriptions were read and coded by the first author, which provided familiarisation and data immersion. Using recommended procedures, systematic analysis of transcripts was undertaken in NVivo 12.0, as the six‐phase, reflexive thematic analysis of interview data^21^, ^22^, ^23^ framed by existing theories and literature, rather than deriving theories only from the data.^24^ Data items were systematically allocated to emergent codes, followed by themes to reflect staff experiences, the meanings ascribed by them and their social reality.^19^

### 2.7 Positionality of researchers

The female lead author and interviewer, as a psychology/science student researcher, has an extensive business background in stakeholder engagement. The second female author has a PhD in occupational health psychology, is a university lecturer and conducts research focused on workplace issues and ageing. The third female author has a PhD in the experiences of older adults, is a university lecturer and conducts research on the experiences and engagement of older adults. This phenomenological study was based on respect for the meanings attributed to the experiences of participants. The conviction and enthusiasm of participants were indicated by tone, body language, repetition and vigorous agreement with reciprocal rephrasing of their comments. The interviewer was responsive to participants' reported experiences, and this may have influenced interactions, with reflexive responses encouraging further disclosure.^23^, ^24^

## 3 RESULTS

**FIGURE 1:**
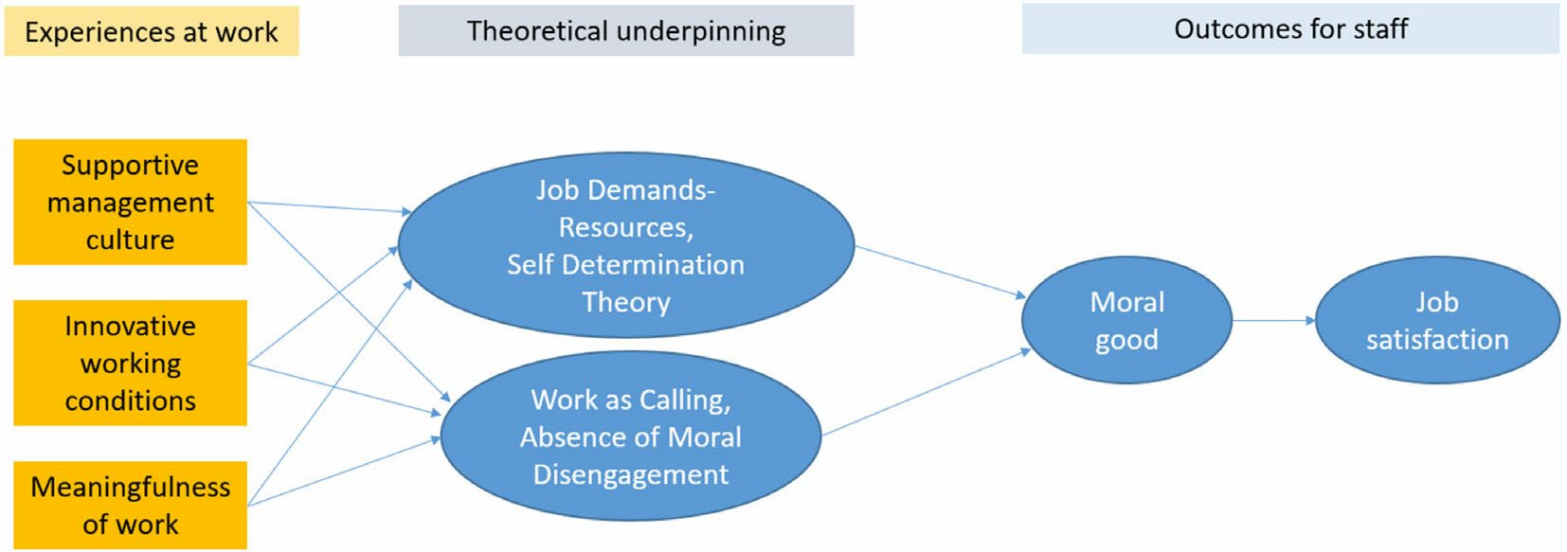
Linkages between experiences at work, the theoretical underpinning and staff outcomes.

The interviews (*n* = 18) resulted in 545 comments, indicating that the research question was effectively addressed by the design of the research, the appreciative inquiry approach and the methods,^25^, ^26^ using interpretive commentary and direct quotes.^24^ The interviews found three themes that demonstrated how staff felt they benefited from the innovations at NewDirection Care. Importantly, more PCC ensured their ability to provide authentic care for residents, showing respect for the dignity of residents, and genuine, enthusiastic enjoyment of their work. The way in which the three themes underpinned positive staff outcomes is shown in Figure 1. Staff spoke about (1) the *supportive management culture*, (2) the *innovative working conditions* and (3) the *meaningfulness* of work. The themes and theories are outlined in Table 1 (as an overview) and in Table 2 (with sample quotes).

**TABLE 1:** Main themes related to job satisfaction, showing the theoretical frameworks and frequency of comments.

Theme	Theory	Codes representing themes	Participants (*n* = 18)	Comments (*n* = 545)
Supportive management culture	JD‐R	Effective leadership	15	49
	SDT	Staff autonomy	14	32
	JD‐R	Sense of community	12	31
	JD‐R	Collaboration and teamwork	12	31
	JD‐R	Hard work/high workload	9	24
	JD‐R	Feel valued by management	8	15
	JD‐R	Relatives at work	7	7
	JD‐R	Friends at work	6	7
	JD‐R	Support from other staff	5	9
	JD‐R	Positive workplace culture	4	13
Innovative working conditions	JD‐R	Better than traditional	16	50
	JD‐R	Pride of working there	14	48
	SDT	Staff enjoy working outside	6	10
	SDT	Staff at the cafe	3	5
Meaningful work	MD*	Making residents happy	16	100
	MD*	Giving dignity to residents	15	65
	WCT	Age care as vocation	12	29
	WCT	This is my ‘dream job’	11	18
	JD‐R	Meaningful work	6	17

Abbreviations: JD‐R, job demands‐resources theory; MD*, absence of moral disengagement; SDT, self‐determination theory; WCT, work as calling theory.

**TABLE 2:** Themes, theories and codes with representative quotations.

Theme	Theory	Codes representing themes and sample quotes from staff	
Supportive management culture	JD‐R	Effective leadership	(P11) ‘the management…, how they've implemented different strategies, and it's worked so beautifully’ (P7) ‘yeah if I go to my manager, with suggestions, like, it happens’
	SDT	Staff autonomy	(P3) ‘it's different yeah, the stuff that you're doing is the same but the way you work is different. Traditional can't do that because they're too task focused, I guess. This is more about personal lifestyle, yes, the tasks have to be completed but there is no real timeframes around them’ (P10) ‘if someone's up, you can go and shower that person, and leave the other person to have a sleep in and things like that. So, yes, I think it's definitely more autonomy’ (P14) ‘it's like we, it kind of feels like we're stepping into our own homes as well. Like, you know, we get to run the house, as we see fit. And there's always so many options. You know, if we've got it done, we can go and enjoy some of the activities with the residents too, and provide a bit extra one on one support when they need it, which is fantastic’ (P17) ‘obviously, we have to meet clinical criteria, what we do with our day is up to us. So, we have autonomy, you need to have you know, have your time management, but don't be time focused’ (P9) ‘…and you're able to just mould your day around them’
	JD‐R	Sense of community	(P2) ‘and the strength for me of that, is um the community engagement for the residents. They're not isolated to just the people that are in here, they're seeing external visitors, children in the community. the residents just having a lot more connection with the wider community. think that's beneficial for the wider community as well, just to have that familiarity with people living with dementia to not see it as that they should be excluded and locked away; they are still active members of the community, engaging, friendly yeah’ (P3) ‘so it's that community feel, like if you're in your own neighbourhood and walking down the street, you're stopping and talking to people’
	JD‐R	Collaboration and teamwork	(P1) ‘the facilities, very friendly, no uniforms, everyone blends in, everyone pitches in where they're needed, you know managers pitch in when staff are away’
	JD‐R	Hard work/high workload	(P6) ‘I always say I love my job, but I do say it is very full on… yeah it is exhausting sometimes’ (P5) ‘so I guess it's just trying to not get so worked up about all the work and just, you know, taking your time and really think about how you're going to plan out your day, and then it will be a lot easier’
	JD‐R	Feel valued by management	(P15) ‘definitely feel that I'm making a difference’ (P10) ‘I feel like I do make a valuable contribution …. Quite often you're making a rapport relationship with people just passing them by’
	JD‐R	Relatives at work	
	JD‐R	Friends at work	(P9) ‘truly, yeah, because, I mean, you create such important friendships, and you know that to be able to have that trust and that, that support around you, you have to have that based on a friendship. But genuinely, there's that, that solid foundation, between everyone’
	JD‐R	Support from other staff	(P1) ‘there is always a lot of support when you need it, you just need to ask. There will always be people to help’ (P9) ‘and then if it got too much, obviously, you call out for support, and someone's there, so and you can always rely on your neighbours’
	JD‐R	Positive workplace culture	(P1) ‘they were happy they were joking when they walked into work, I hadn't seen that too often. Especially there were agency staff, so people who aren't regularly positioned, and they were happy to come in, they'd have a chat with reception and then they would get on to work. I know a lot of the management try to keep that positivity, because it is a very high burnout industry, people get… people take time off, they get burnt out, they get stressed and they want to leave, so if you can try and make it as positive, positive as a place to work, people would want to stay. Even the management team, if they're low on staff, will be working on the floor. we fill in where we're needed, and we're expected to be able to do that wherever possible because it helps everyone out’ (P2) ‘I think part of being innovative and new and different they're very accepting of new ideas. here they will listen and say oh how could we do it differently? do you have any suggestions what's working what's not working can we change it?’
Innovative working conditions	JD‐R	Better than traditional	(P12) ‘people in traditional don't have the same level of freedom. They, yeah, they basically are forced to be part of a routine. Here they get to choose when they have their meals, when they get up, what time they do their things’ (P18) ‘traditional aged care is very task focused’
	JD‐R	Pride of working there	(P16) ‘where else would you go if want them to thrive?’ (P4) ‘I tell them that I work in a quite a, what's the word for it, quite a unique aged care facility, they're very focused on making people feel at home’
	SDT	Staff enjoy working outside	(P13) ‘yep, I think it's just a beautiful place to relax for a moment because it's tranquil. And I just sit down there with the residents sometimes. Yeah, and then when you go from one house to another, it feels like you're just going for a walk. It doesn't feel like you're going from one job to another. I really enjoy the amount of green space yes and more than 50% green spaces’
Meaningful work	MD*	Making residents happy	(P10) ‘we might be (at the café) with certain residents, but then we'll have other residents come and say hi. Want to sit down with us, so we encourage that, yeah. And so, we have a whole big group at the table, and we get the ones we're with to socialise as well. So, you know, it's like a big gathering, there is a lot of happiness here. It's more about, we're part of the family, and that's how I see it, yeah, we're family’ (P15) ‘because it's such a non‐restrictive model, I think our residents are more happy’ (P2) ‘yes well happy residents make happy staff, I think. It is that yeah, you're delivering care and they're thankful for it, rather than being resistive yeah’ (P5) ‘you know that relationship that you have with the residents. I think that really helps, you know, you actually sitting down and taking the time for that resident. I feel like they feel like we're involved with them and actually care for them… So, I believe that makes the residents happy, and it also makes the staff happy, with the residents being happy. They really encourage, you know, if a resident wants you to sit down and have a coffee with them, yes, you're allowed to go sit and have a coffee and chat’
	MD*	Giving dignity to residents	(P11) ‘it's, it's the way we care for our residents, the empathy that we have, the respect that we give, these are all the little things that we do, that, again, validate our residents' existence. Yes, that's where we are different. We are genuinely caring people, yeah, but to understand and also to, if you're in a community that has people with cognitive impairments, you're going to have conversations with them that don't make sense, but that's okay. Live in their world, try and see what they see. Just live in their world. Still give them the respect and the dignity to still validate what they're saying’ (P11) ‘the dignity at all times to the, even down to the point that when our residents pass away, they leave through the front door through a guard of honour of residents… This person has been part of our lives as well, … so there again, it's giving people dignity and giving them respect’ (P5) ‘yeah, yeah. And even with the residents, like here, if I've got a 98‐year‐old man that nearly every week, he'll come and see me. Hey, can I use the lawnmower? Yep, no, no problem, mate. I'll start it up for him. And he goes and mows as many lawns as he wants…dignity of risk’
	WCT	Age care as vocation	(P14) ‘I love working with the residents. I tell them that I absolutely love it. just getting to sit in the gardens and chat with them is, it's one of my favourite things. You care a lot, and you want to do better, and you want to make people's lives easier and better. You know, it's not just a job’ (P15) ‘so you need to have your heart into it. you know, in honesty, yes, aged care is very hard, but you do it, you do it to it's like, it's like a contribution to the society. It's almost, I do get paid to do something I enjoy, so it's a bonus in that way’ (P16) ‘really, I found my spot, found my home, found my calling. Yeah, I love them. Doesn't feel like a job. It feels like you're going to Grandma's house to do something for the day’
	WCT	This is my ‘dream job’	(P10) ‘I walk in here and I love my job; I love walking into the environment. I do. It's beautiful. It's really a good place to work.’ (P2) ‘whereas here it is not a role that I expected to enjoy, and yet I have way more job satisfaction, and a better work life balance’ (P9) ‘I absolutely love it. Best job I've ever had in my life’
	JD‐R	Meaningful work	(P12) ‘and I love my work because it involves being engaged with residents. And it's very fruitful and meaningful’ (P14) ‘you know, it's that they're not being punished, essentially, for having something that they can't control, which I absolutely love… It just, it feels like everyone cares about everyone, no matter what… It's lovely, feeling good when you're working, even on the hard days’ (P1) ‘I have way more job satisfaction, and a better work life balance’

Abbreviations: JD‐R, job demands‐resources theory; MD*, absence of moral disengagement; SDT, self‐determination theory; WCT, work as calling theory.

As shown in Tables 1 and 2, the *supportive management culture* was theoretically best understood by the JD‐R theory, with SDT also contributing. All participants commented on the job resources that made the workplace culture very positive, such as effective leadership, a strong sense of community, collaboration and teamwork, and being able to work with family and friends. The strong sense of community amongst staff showed the importance of leadership to foster collaboration and social supports amongst staff, which built resources to manage the job demands associated with the workload of caring for frail or older adults.

*Innovative working conditions* were represented by the JD‐R and SDT again, although this was more captured by being ‘Better than traditional’. The results indicated that innovative models of care can be more resource‐rich than traditional models of care, and resources from affective well‐being, such as being proud and enjoying work, were also important for the satisfaction and retention of staff. The final theme, the *meaningfulness of work*, represented the absence of moral disengagement, seeing work as a calling and meaningfulness as a job resource (the JD‐R). Being able to work in ways that gave happiness and dignity to residents, along with work being considered a vocation and a ‘dream job’, showed that the value employees place on their work was important to job satisfaction. Such work allowed employees to fulfil their personal moral obligation to provide the best possible care for everyone in their care, by focusing on residents, not themselves.

The quotes in Table 2 demonstrate the depth of staff satisfaction with their work, their appreciation for sufficient resources (e.g. flexibility and supportive management) and the importance of this as morally good work. Staff consistently endorsed an authentic, strongly person‐centred care ethos and the ability to support the autonomy and dignity of residents, as motivating staff and engaging residents.

## 4 DISCUSSION

The current qualitative study explored working conditions for staff at NewDirection Care, which provides innovative residential aged care, finding that staff were consistently and enthusiastically positive about their work, with three themes emerging from the interviews: (1) supportive management culture, (2) innovative working conditions and (3) the meaningfulness of work. The themes provided strong evidence of the ways in which innovation can increase job satisfaction and retention of staff. The combination of organisational context with the built environment, and novel and flexible work roles greatly strengthened job satisfaction, and the findings demonstrated the importance of a whole‐of‐organisation approach in the processes of changing models of care. The study also highlighted the importance of theoretical models that can explain how innovation could be applied in other residential aged care homes and how attending to organisational structures can support and encourage more positive outcomes for staff, and the care they provide to residents.

The innovations at NewDirection Care demonstrate how models of aged care can align with the Royal Commission's^3^ recommendations for residential care, for example, having small‐scale houses. More than just being about the size of the household, however, the innovations have also expanded the setting for cottage living to encompass the areas that would usually accompany homes (such as garden sheds, pathways and chicken coops) and the familiar urban setting for homes (such as shops and amenities). As part of the extensive innovations of the MicroTown™ at NewDirection Care: Firstly, the design of the built environment gives residents access to on‐site allied health, ensures that people with cognitive impairment are not segregated, and residents live in non‐institutional housing; and secondly, the design of work means staff have autonomous, rather than task‐focused, working conditions. From the interviews, it was clear that all these innovations created meaningful and satisfying working conditions that could be theoretically explained. The themes about management culture and innovative work tasks related particularly to the way in which work is organised. Staff experiences were framed by jobs with sufficient resources to care for older residents, rather than by the demands of that workload (as the JD‐R theory^9^, ^10^), where they had responsibility/autonomy for their work schedules (as SDT^11^, ^12^), where work centred on making residents happy and maintaining their dignity (as the absence of MD^13^, ^14^), and where work was a vocation that was deeply meaningful and worthwhile (as WCT^15^, ^16^).

Interestingly, about half the sample had previous experience in traditional residential aged care homes. These staff discussed the strong contrast between traditional homes and the model at NewDirection Care, with their current employment providing more caring leadership, more meaningful living conditions for residents and being better than traditional models of care, although new organisational structure did require adjustment. Importantly, staff believed sincerely in the innovative ethos, felt supported and encouraged by management, and they greatly valued ‘making residents happy’ and ‘giving residents dignity’ (as high levels of PCC), and the consequent empowerment of residents. A recent observational study in the Netherlands^27^ showed that innovations in residential aged care homes must consider organisational cultures (such as values and competencies), as well as long‐term effects of changing the physical environment and models of care. Transitioning from traditional models to innovative models or new conditions may be stressful, due to gaps in understanding^27^ or resistance to adapting to different ways of providing care.^28^ More thoughtful or careful selection of new employees and appropriate training for new roles had eased these issues in the current study, reflecting positive workplace culture and effective leadership.

Once working, staff experiences were framed by their resource‐rich jobs (e.g. authentic leadership from management, supportive colleagues, pleasant environment and pride in work) that mitigated their workloads for caring for older residents. The current study supported the importance of the JD‐R theory^9^, ^10^, ^29^ to explain positive staff outcomes, the benefits of sufficient resources to manage their demands^29^ and to show that resources arise at each level of the organisation^10^: from management, with fellow staff, to the work itself. The comments for each theme clearly demonstrated how intertwined the resources from each level were (e.g. effective leadership and teamwork), and how these increased job satisfaction and enthusiasm for work.

Resource‐rich jobs also have the consequence of increasing personal autonomy, as staff could manage their days to be responsive to residents' needs, thus meeting their own psychological needs (e.g. to have their need for autonomy supported, not frustrated) as outlined by SDT.^11^, ^12^ The findings of the current study also support a recent systematic review of 28 studies of aged care nurses^30^ where job satisfaction was increased by staff experiences of self‐determination, psychological empowerment and autonomy. In the current study, autonomy satisfaction^11^, ^12^ also came from freedom within the outdoor setting, community engagement, self‐actualisation and trust to complete tasks.

The meaningfulness of work, the final theme of the interviews, captures the value staff placed on the work they did for other people, expressed by interviewees as *making residents happy* and seeing them smile. The positive benefits of working in an authentic, resident‐centred way are similar to those reported in Netherland's green‐care farms.^7^ The current study shows that meaningful work is based on the absence of moral disengagement^13^ (e.g. respect and dignity of older adults is protected by action and language, and lack of cynicism). Therefore, moral working conditions are maintained by genuinely enabling person‐centred care above profitability, which gives dignity to residents and guards against cynicism about management.^14^ Meaningful tasks were also mentioned, where working in aged care was seen as a valued vocation and a ‘dream job’.^15^, ^16^ By emphasising the good they do and seeing the benefits they themselves bring to the lives of residents, staff can see the meaningfulness of what is achieved and can regard aged care as a ‘calling’.^15^, ^16^ Personal care workers are the primary interface between the authentic management ethos and the treatment of residents. However, in the current study, even staff employed in other roles (e.g. maintenance and administration) were encouraged to contribute to residents' well‐being. In contrast to previous research,^5^ which found staff were dissatisfied when they did not have time to provide holistic, meaningful care, all staff in the present study noted they were encouraged to spend time with residents; for example, they were permitted to stop work and have a coffee at a resident's invitation, rather than solely focusing on completing specified tasks.

Overall, the interviews showed how the work structure allowed staff to have autonomy over their work, feel competent in doing so, build relatedness by making residents happy and experience belongingness in friendships and teamwork. Our data indicated that valuable, morally important work, in line with moral engagement, is shown in giving happiness and dignity to residents. From participants' comments, it is clear this leads to strong commitment to continuing in the work and caring for residents and to staff satisfaction.

### 4.1 Alternative reasons for results

The positivity of responses may reflect individual differences of the staff (e.g. more optimistic or enthusiastic by nature), differences in the residents in their care (e.g. mostly ambulatory residents requiring reduced care) or social desirability bias (e.g. staff may have wanted to garner favour with their employer by only reporting positive experiences). The research team may have given more positive interpretations of interviews in light of the innovations, rather than a more cautious or sceptical interpretation.

### 4.2 Limitations, strengths and future research

Limitations of the research include, firstly, interviews were conducted with staff who volunteered, so we may not have interviewed those who disagreed with the aged care home's ethos. Secondly, analysis of the interviews may be subject to the biases of the research team, and lastly, randomised sampling was not possible. As volunteers came from one unique site, these findings may not be transferrable to other residential aged care homes with different models of care. The research strengths were consistency of staff experiences regardless of organisational roles, with data saturation quickly achieved and demonstrated across work roles. Further qualitative and quantitative studies are recommended for both innovative and traditional residential aged care homes to understand antecedents and consequences of job satisfaction, emphasising motivations, values and organisational structures that sustain meaningful work and retention of aged care staff. Particularly, it would be valuable to learn if there are types of innovations (e.g. built environment or organisational ethos) that are most influential for staff satisfaction, which could be retrofitted into existing residential aged care homes.

## 5 CONCLUSIONS

In conclusion, the uptake of innovative models of aged care requires careful consideration and planning around the extent of organisational issues likely requiring change, such as updating Human Resources policies, rethinking job role descriptions, how managerial oversight and control of day‐to‐day operational matters is enacted, the ways in which staff are selected and trained, as well as the physical environment in which care is provided. The commitment of substantial time and financial resources to achieve successful transition to innovative models of care would therefore require interested organisations and aged care providers to consider what innovation would or could look like in their particular economic situation and geographic location. The aged care home in the current study represents the culmination of the organisation's extensive in‐house exploration and study of best‐practice examples and early trials of design ideas. The current study does not provide an economic rationale for their model but shows clearly the theoretical frameworks that can be used to understand positive workplace experiences of the staff who work there, and what factors within an organisation's control can build staff morale, maintain job satisfaction and increase likely retention of valued staff. By identifying factors contributing to staff satisfaction, the current study highlighted areas for exploration by developers and operators of residential aged care homes to provide PCC and address the long‐term, critical shortage of aged care staff to give our ageing population access to the best possible care.

## CONFLICT OF INTEREST STATEMENT

No conflicts of interest declared.

## ETHICS STATEMENT

This study was approved by the Human Research Ethics Committee, University of the Sunshine Coast (Ref: S241972) in line with the Declaration of Helsinki.

## Data Availability

Data are available upon reasonable request.
